# Intravenous Cavutilide for Pharmacological Conversion of Paroxysmal and Persistent Atrial Fibrillation in Patients with Heart Failure

**DOI:** 10.3390/jcdd10120487

**Published:** 2023-12-06

**Authors:** Maria M. Beliaeva, Khava M. Dzaurova, Yulia A. Yuricheva, Peter S. Novikov, Nikolay Yu. Mironov, Gennady S. Tarasovskiy, Maksim A. Zelberg, Sergey F. Sokolov, Sergey P. Golitsyn

**Affiliations:** Federal State Budgetary Institution, National Medical Research Centre of Cardiology Named after Academic E.I.Chazov of the Ministry of Health of the Russian Federation, Academician Chazov str., 15a, Moscow 121552, Russia

**Keywords:** heart failure, atrial fibrillation, rhythm control, cardioversion, antiarrhythmic drugs

## Abstract

This work aimed to study the efficacy and safety of the class III antiarrhythmic agent cavutilide (Niferidil, Refralon) for pharmacological cardioversion in patients with paroxysmal and persistent atrial fibrillation (AF) and heart failure (HF). Methods and Results: In this retrospective cohort study, 58 patients with stable HF (aged 69 [61;73] years, 30 males, 78% with persistent AF) and 274 patients without HF (aged 63 [57;70] years, 196 males, 56% with persistent AF) were included. The median AF duration in the group with HF was 35.5 [10.6;124] days, and that in the group without HF was 14.5 [3.6;90] days. All patients received 5–30 µg/kg cavutilide intravenously in one to four (if needed) boluses of 5–5–10–10 µg/kg at 15 min intervals. Subsequent boluses were not administered if the patient’s sinus rhythm (SR) was restored or if bradycardia, QT prolongation > 500 ms or evidence of proarrhythmia was observed. Holter electrocardiogram monitoring was started before infusion and was continued for 24 h. The main criterion for an antiarrhythmic effect was sinus rhythm restoration within 24 h of the initial bolus. Results: Cavutilide converted AF to SR in 37.9% of patients with HF after bolus 1 (5 µg/kg), in 58.6% after bolus 2 (cumulative dose = 10 µg/kg), in 74% of cases after bolus 3 (cumulative dose = 20 µg/kg) and in 92.8% of cases after bolus 4 (cumulative dose = 30 µg/kg). Cavutilide was effective in 89% of cases with persistent AF with a median duration of 70.5 [30;159] days and in 92% of cases with paroxysmal AF with a median duration of 36 [24;102] h. In the group of patients without HF, the effectiveness of bolus 1 was 36.9%, that of the bolus 2 was 58%, that of the bolus 3 was 77% and that of the bolus 4 was 90.1%. Cavutilide restored SR in 90% of patients with persistent AF with a median duration of 82.5 [28;180] days and in 90% of cases with paroxysmal AF with a median duration of 50 [24;120] h. No statistically significant difference in the probability of SR restoration or the effectiveness of each bolus of cavutilide was found between patients with and without HF. The median time to restoration of SR in patients with HF was 23 [11;55] min, and that in patients without HF was 22 [10;45] min (*p* = 0.424). No cases of symptomatic/severe bradycardia were observed in either group. QT prolongation over 500 ms after cavutilide injection was registered in 19% of patients without HF and in 15.5% of those with HF (*p* = 0.58). Short runs of Torsade de pointes tachycardia occurred in one patient (0.4%) without HF after 10 µg cavutilide administration and were successfully treated with MgSO_4_. Conclusions: Cavutilide demonstrated a high likelihood of AF conversion to SR in paroxysmal (92%) and persistent (89%) arrhythmia and HF. Concomitant HF and its severity do not affect the efficacy and safety of cavutilide.

## 1. Introduction

The prevalence of heart failure (HF) has grown at an alarming rate in the last decade. According to data from the American Heart Association (AHA), about 6 million patients with HF are registered in the USA [1,2]. According to epidemiological studies in the Russian Federation, HF occurs in 7% of the country’s total population [3]. Healthcare costs for treating patients with HF exceed billions of US dollars and are constantly growing annually.

The progression of various diseases and their concomitant forms leads to the development of HF. However, coronary heart disease (CHD), arterial hypertension (AH), congenital and acquired heart diseases, cardiomyopathies and arrhythmias remain the leading causes of this clinical syndrome [4]. 

Arrhythmias play a special role in HF pathogenesis. On the one hand, heart rhythm and conduction disorders may lead to HF; on the other hand, the progression of underlying cardiovascular disease and HF may cause arrhythmia onset and worsen clinical prognosis.

Atrial fibrillation (AF) is the most common atrial arrhythmia associated with HF. The percentage of patients with HF suffering from AF increases with patient age and the duration of HF. This fact is due to structural myocardial remodeling, neurohormonal activation and a decreasing left ventricle (LV) contraction function. The onset and progression of AF in patients with HF are associated with a worse prognosis due to circulation decompensation, an increased risk of stroke and, as a result, an increased mortality rate in this population [4]. 

Currently, electrical cardioversion (ECV) remains one of the main treatment options for hemodynamically unstable AF patients and patients with acute decompensation of HF. If the patient’s condition is stable, pharmacological cardioversion may be considered. Most antiarrhythmic drugs effectively stop recent-onset AF and atrial flutter (AFL). In cases of persistent AF/AFL, the efficacy of AADs is significantly reduced. Ibutilide is one of the most effective pharmacological agents in cardioverting persistent AF, resulting in a 48% sinus rhythm restoration rate for an AF/AFL duration of 30 days [5]. According to clinical studies, vernakalant successfully cardioverted AF with a duration of 7 days or less in 47–52% of patients, but it was successful only in 8% of patients with an AF episode duration of 8–45 days [6,7,8,9]. Furthermore, vernakalant is ineffective in patients with AFL [9]. Thus, the currently available data show that there are no effective drugs for pharmacological conversion when an episode of AF lasts more than 7 days. 

Cavutilide (Refralon, Niferidil, RG-2) is an original class III antiarrhythmic drug (AAD). Cavutilide induces concentration-dependent blockade of the delayed rectifier potassium current (IK) and inhibits the L-type calcium current (ICaL). This leads to an extension of the repolarization phase of the action potential and to an extension of the refractory periods of cardiomyocytes. Cavutilide does not significantly affect sinus node automaticity, and it has no effect on the conduction system of the heart, including the atrioventricular node [10]. On an electrocardiogram (ECG), cavutilide induces a moderate prolongation of the QT/QTc interval without significant changes in other ECG parameters.

When used in doses up to 30 μg/kg, cavutilide demonstrates high antiarrhythmic action (up to 91.6%) in restoring sinus rhythm in patients with persistent AF/AFL, with a moderate risk of proarrhythmic effects (up to 1.7%) [11,12,13]. 

Currently, cavutilide is included in the Russian national clinical guidelines and the Eurasian clinical guidelines for the diagnosis and treatment of atrial fibrillation and flutter. The drug is widely used in the standard of care for managing patients with persistent and long persistent AF [14,15].

Due to cavutilide’s antiarrhythmic efficiency and low percentage of adverse effects in AF/AFL, we studied the drug’s effectiveness and safety profile in patients with AF/AFL and chronic heart failure. 

## 2. Methods

This retrospective multicenter cohort study included patients with symptomatic paroxysmal or persistent forms of AF who underwent pharmacological cardioversion with cavutilide in a hospital setting. All patients were 18 years or older and had indications for sinus rhythm (SR) restoration. Depending on the presence or absence of HF, patients were divided into two groups.

Study exclusion criteria: congenital or acquired QT interval prolongation >440 ms at the time of cardioversion; bradyasystolic AF/AFL (mean heart rate <50 bpm or >3 s pauses); sinus bradycardia, sinoatrial blockade in history; atrioventricular block of any degree; evidence of previous AAD proarrhythmic action, acute myocardial infarction (MI); acute period after coronary artery bypass grafting or other cardiac surgery; decompensated or severe HF; hyperthyroidism or decompensated hypothyroidism; uncorrectable electrolyte disorders (potassium level less than 3.5 mmol/L); blood clots in the heart cavities or grade III–IV spontaneous echo contrast observed with transesophageal echocardiography; indications for emergency ECV due to patient’s hemodynamic instability.

According to the CHAD2S2-VASc score, all patients received long-term anticoagulation before and after cardioversion.

Cardioversion with cavutilide was performed in a cardiac intensive care ward under 24 h of continuous ECG monitoring. Cavutilide (0.1% solution) was administered intravenously for 2–3 min in three (allowed to divide the first bolus into two consecutive injections: 5 + 5 μg/kg of body weight) consecutive boluses of 10 mg/kg of body weight each at 15 min intervals. After each i.v. bolus and before the subsequent administration, the ECG parameters were determined and clinical assessment of the patient was performed. Holter electrocardiogram monitoring was initiated before infusion and was continued for 24 h. The criteria for stopping i.v. administration of cavutilide were as follows: restoration of SR; deceleration of heart rate below 50 bpm; QT/QTc prolongation ≥500 ms; proarrhythmic effects (occurrence of sustained ventricular tachycardia (VT) or non-sustained VT, including Torsade de pointes).

Primary endpoints were the incidence of SR restoration within 24 h in patients with persistent AF/AFL after the first dose of cavutilide and the incidence of SR maintenance in patients 24 h after the first cavutilide dose.

### 2.1. Measurement of Electrocardiographic Parameters

ECGs were obtained to evaluate the heart rhythm, atrial activity, R-R and QT/QTc interval before, during and 24 h after each bolus of cavutilide. The QTc interval in AF patients was measured as the QT interval after the shortest and longest R-R intervals (in a 10 s ECG strip)/the square root of the preceding R-R interval. The average QTc of these was used as the adjusted QTc interval. The QT interval in SR was measured according to Lepeshkin and Surawicz’s method [16]. The Bazett QTc formula was used for QT rate correction.

### 2.2. Statistical Analysis

Continuous variables with Gaussian distributions were described as mean and SD (M ± SD) and continuous variables with non-Gaussian distributions as median, lower and upper quartile (Me [Q_1_;Q_3_]). The χ2 test was used to compare discrete variables. Continuous variables were analyzed using Student’s *t*-test or the Mann–Whitney test, as applicable. A *p* value < 0.05 was considered statistically significant. Analysis of variance was used to test the difference between the mean of several subgroups of variables. All the statistical calculations were performed using StatTeh v.3.1.8 and the Statistica 64 version 12.5.192.0.

### 2.3. Legal Aspects

Cavutilid is a registered drug in the Russian Federation and was used in full accordance with the approved instructions for its medical use. Due to this approval, permission from the National Center for Expertise of Medicinal Products and the National Ethics Committee was not required to conduct this study.

All included patients indicated informed consent to pharmacological cardioversion.

### 2.4. Limitations

As stated above, this study is retrospective in nature, and it did not allow us to form a control group or to perform sample size calculations for patients with HF at the stage of study planning. So, the second limitation was the relatively small sample study size.

## 3. Results

Three hundred thirty-two patients with paroxysmal and persistent AF were included in this retrospective multicenter study. Fifty-eight patients had stable HF (without episodes of decompensation during the last 3 months) (aged 69 [61;73] years, 30 males). Six of them were classified with New York Heart Association (NYHA) class I symptoms, forty-four with NYHA class II symptoms and six with NYHA class III symptoms. Patients with NYHA class III symptoms or LVEF under 40% had clinical worsening only during an episode of AF. Forty-five (78%) patients had persistent AF and thirteen (22%) had paroxysmal AF. The HF group included patients with preserved, mildly reduced and reduced EF (Table 1). 

The second group consisted of two hundred seventy-four patients without diagnosed HF (aged 63 [57;70] years, 196 males). All of them had preserved EF with a median of 60 [55–60]%. A total of 153 (56%) patients had persistent AF and 121 (44%) had paroxysmal AF. The clinical characteristics of the study patients are summarized in Table 2 and Table 3.

Compared to those without HF, patients with HF were older (69 vs. 63 yr; *p* = 0.023), had a higher CHADS2-VASc score (3 vs. 2; *p* < 0.001) and were more likely to have CHD (*p* < 0.001), a history of MI (*p* = 0.03) and diabetes mellitus (*p* = 0.001). Also, they had a lower mean ejection fraction (57% vs. 60%. *p*< 0.001), a larger left atrium volume (85 vs. 78 mL; *p* = 0.004) and a higher left ventricle mass index (100% vs. 87%. *p* < 0.001). 

Patients with HF in common also had a longer current AF/AFl duration (35.5 vs. 14.5 days. *p* = 0.03).

There were no significant differences in treatment strategies between the groups, other than more frequent use of diuretics and mineralocorticoid receptor antagonists among those with HF.

In spite of all these factors, there were no significant differences in the efficacy and mean required doses of cavutilide.

**Figure 1:**
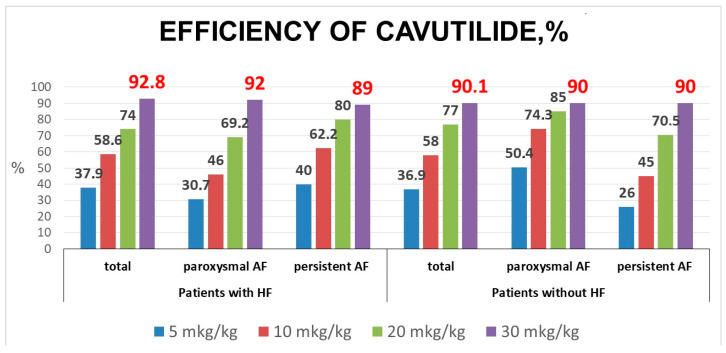
Efficacy of cavutilide (total and in doses) among patients with different forms of AF and with or without heart failure. AF—atrial fibrillation; HF—heart failure.

The first bolus of 5 µg/kg cavutilide restored SR in 22 (37.9%) of 58 patients. The second 5 µg/kg bolus (cumulative dose = 10 µg/kg) converted AF to SR in 12 patients. Thus, 10 µg/kg total cavutilide was effective in 34 (58.6%) patients. The third bolus of 10 µg/kg intravenous cavutilide restored SR in nine patients and the cumulative efficacy of 20 µg/kg cavutilide was 74%. The fourth bolus of 10 µg/kg cavutilide converted AF to SR in nine patients. The overall efficacy of 30 µg/kg cavutilide in the group of patients with HF was 92.8% (Figure 1).

Cavutilide was effective in 40 of 45 (89%) persistent AF cases with a median duration of 70.5 [30;159] days and in 12 of 13 (92%) cases of paroxysmal AF with a median duration of 36 [24;102] h.

In the group of patients without HF, the first bolus was effective in 101 (36.9%) patients. The second bolus was effective in 58 patients (cumulative efficacy was 58%). The third bolus restored 52 patients with an overall success rate of 77%. Finally, the fourth bolus converted36 patients to SR. Thus, total efficacy of 30 µg/kg cavutilide in the group of patients without HF was 90.1% (Figure 1).

Cavutilide restored SR in 138 of 153 (90%) patients with persistent AF with a median duration of 82.5 [28;180] days and in 109 of 121 (90%) paroxysmal AF cases with a median duration of 50 [24;120] h.

**Figure 2:**
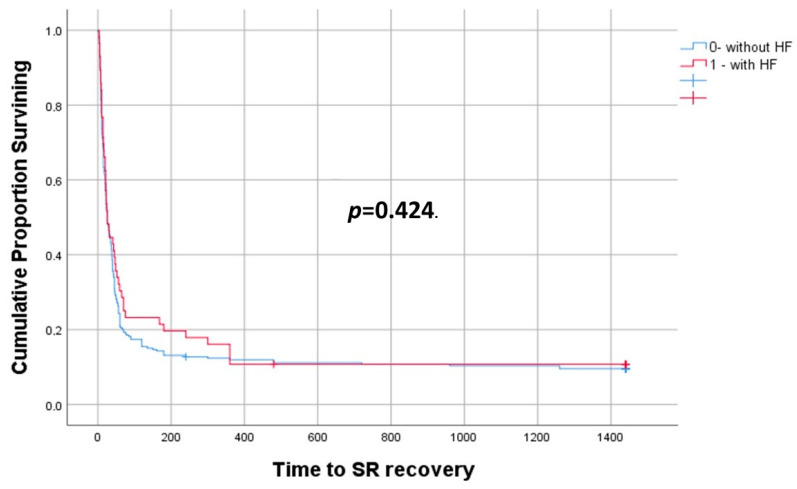
Time to SR recovery after start of cavutilide infusion, comparing those patients with and without HF. *p* = 0.424.

There was no statistically significant difference in the probability of SR restoration and cavutilide’s bolus-dependent effectiveness between patients with and without HF (Figure 2).

The median time to restore SR from the start of i.v. infusion in patients with HF was 23 [11;55] min and, in patients without HF, was 22 [10;45] min (*p* = 0.424) (Figure 2).

**Figure 3:**
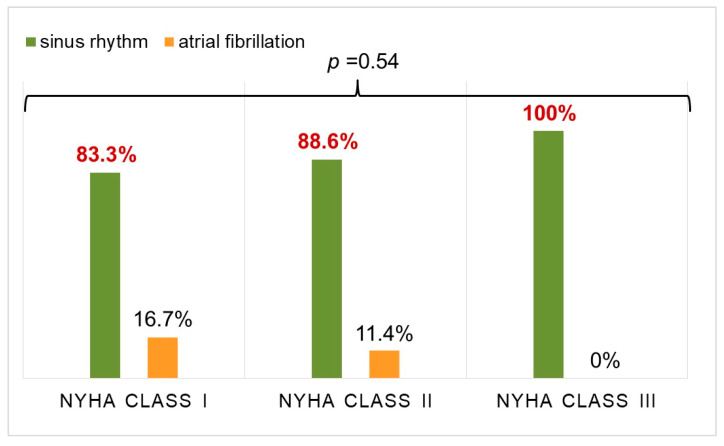
Efficacy of cardioversion with cavutilide depending on the NYHA class of heart failure.

There were no significant differences in cavutilide’s efficacy between patients with NYHA class I, II and III symptoms (Figure 3).

Among patients with HF, SR was maintained during the 24 h of observation in 84.6% of responders after terminating paroxysmal AF and in 84.4% after converting persistent AF. 

**Figure 4:**
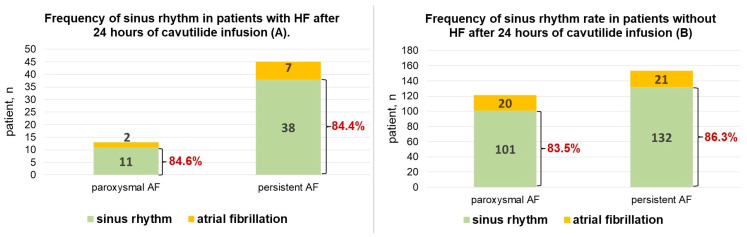
(**A**)—Frequency of maintaining sinus rhythm in patients with heart failure and different forms of atrial fibrillation after 24 h of cavutilide infusion. (**B**)—Frequency of maintaining sinus rhythm in patients without heart failure and different forms of atrial fibrillation after 24 h of cavutilide infusion. HF—heart failure; AF—atrial fibrillation.

In the group of patients without HF, maintaining SR after cardioversion was observed in 83.5% of paroxysmal AF cases and in 86.3% of persistent AF cases (Figure 4).

No correlation was found between AF duration (r = −0.024. *p* > 0.05), LA volume (r = 0.13), LVEF (r = 0.17) and cavutilide’s effectiveness (*p* > 0.05). Multiple regression analysis also did not demonstrate a significant effect of the presented parameters on the effectiveness of cavutilide.

Only body mass index (BMI) had a slight correlation with the effectiveness of cavutilide cardioversion (r = −0.15, *p* < 0.05). Specifically, patients with ineffective SR restoration after cavutilide’s administration had significantly higher BMI values (29.4 [26.1;33.2] vs. 33.2 [29.1;35.8], *p* = 0.010).

HF presence and NYHA class of HF (*p* = 0.54) did not influence the rate of SR restoration and maintenance (Figure 3 and Figure 4).

### Cavutilide Side Effects and Electrocardiographic Findings

**Figure 5:**
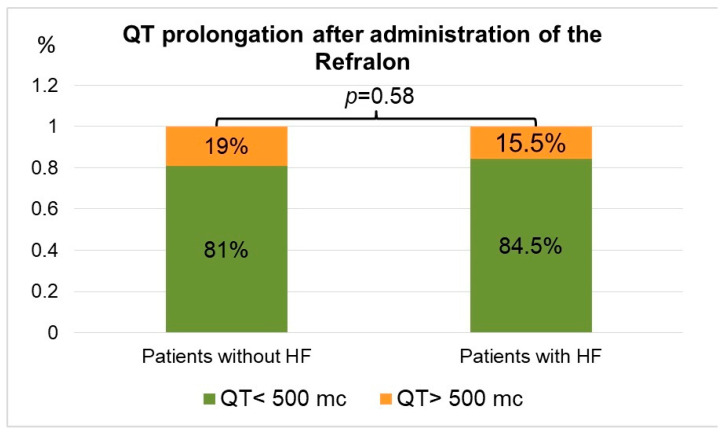
Percentage of QT prolongation >500 mc in patients with and without HF after cavutilide boluses. HF—heart failure.

Analysis of cavutilide’s influence on the duration of the QT interval as the main safety parameter of class III antiarrhythmic agents was performed. No difference in the frequency of absolute values of QT exceeding the potentially dangerous value of 500 ms was found between patients with HF and without it (*p* = 0.58) (Figure 5).

Among 23 patients with HF, 9 (15.5%) patients had a QT interval over 500 ms. However, HF severity did not correlate with maximal QT duration (*p* = 0.18).

**Figure 6:**
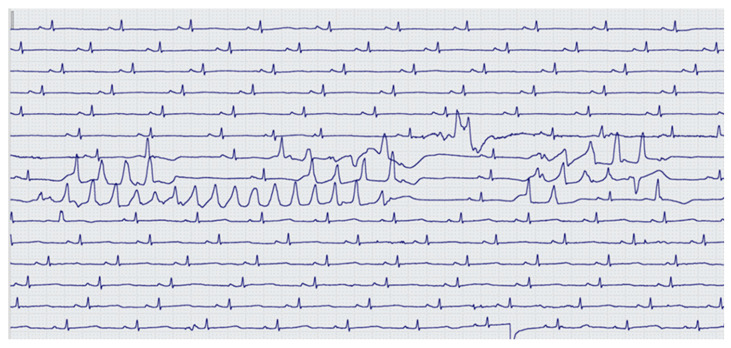
Episode of non-sustained typical polymorphic VT (Torsade de pointes) in a female patient without HF after 10 µg cavutilide administration.

In the group of patients without HF, QT exceeded 500 ms in 50 (19%) cases. One patient (0.4%) in this group after 10 µg cavutilide administration and SR restoration had QT interval prolongation up to 560 ms and an episode of non-sustained typical polymorphic VT (Torsade de pointes). This patient was successfully treated by MgSO_4_ injection (Figure 6).

**Figure 7:**
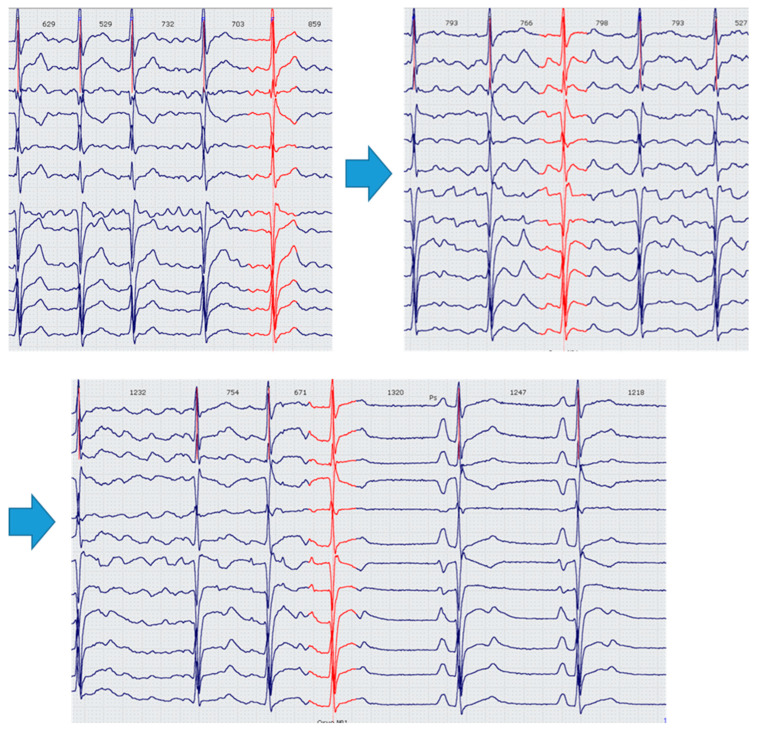
Transformation of irregular high-frequency AF waves into regular low-frequency AFL waves and subsequent SR restoration after cavutilide infusion.

Typical in cavutilide administration, transformation of irregular high-frequency AF waves into regular low-frequency AFL waves (mostly atypical flutter) has been observed more often in groups of patients with HF (22% vs. 14% *p* = 0.04) (Figure 7). However, this phenomenon did not influence the administered cavutilide’s effectiveness and did not lead to deterioration of hemodynamics.

Cavutilide’s administration did not cause a significant HR decrease during persistent AF. After SR restoration, 1.7% patients with HF and 3.5% without HF had sinus node pauses >3 s (*p* = 0.69). Sinus bradycardia <50 beats per minute developed in 3.4% of cases with HF and in 7.4% of cases without HF (*p* = 0.27). The minimal heart rate after SR restoration was 47 bpm in both groups.

In all cases, bradycardia was asymptomatic and did not require i.v. atropine nor external pacing.

## 4. Discussion

Previously, restoration of SR was considered primarily as a symptomatic treatment due to research data, demonstrating the equivalence of the “rhythm control” and “rate control” strategies. However, the results of large prospective multicenter study EAST-AFNET convincingly proved the advantage of an early (within a year after diagnosis of AF) “rhythm control” strategy, due to reductions in cardiovascular mortality, risk of acute cerebrovascular complications, hospitalization for acute coronary syndrome and HF decompensation [17]. 

The efficacy of all currently approved antiarrhythmic drugs for pharmacological cardioversion of AF sharply decreases in patients with persistent AF. In many cases, ECV remains the only effective way to restore SR but may be associated with various complications caused by sedation (laryngospasm, aspiration, hypoventilation, etc.), electrical trauma, sinus node arrest after ECV, etc. 

The advent of the new antiarrhythmic drug cavutilide has greatly expanded the medical options for management of paroxysmal and persistent forms of AF.

Cavutilide with a three-step dose i.v. administration (10 to 30 μg/kg) showed a high efficacy and safety profile in managing persistent and paroxysmal forms of AF compared to ECV [18,19]. Moreover, according to a recent study, SR recovery was achieved in 22 (84.6%) of 26 patients with persistent AF, in whom ECV was previously not effective [20]. To our knowledge, this is the first currently available study, demonstrating significant antiarrhythmic efficacy in this category of patients. 

According to previous successful results, we studied cavutilide use in patients with HF and AF. Our study demonstrated no drug-related complications or adverse effects in the group of patients with NYHA class I–III HF, compared to the non-HF group. At the same time, the drug efficacy was comparable with the results obtained among patients without HF events.

In both groups with and without HF, our study demonstrated 74% and 77% SR restoration, during one hour of i.v. drug administration, including persistent forms of AF. As known, amiodarone is the most widely used antiarrhythmic drug for AF/AFL management with an 85% average success rate in restoring SR and mostly has a delayed effect (5–12 h) [20,21]. At the same time, the percentage of successful cardioversions with amiodarone significantly decreases in persistent forms of AF and, in long persistent AF, becomes almost comparable to placebo [21,22,23].

Moreover, results of a blind randomized study comparing refralon (cavutilide) and amiodarone in paroxysmal AF/AFL showed that the effectiveness of the minimum dose of refralon (5 mcg/kg) (56.7%) did not significantly differ from the efficacy of the maximum amiodarone dose (1200 mg/day) (57.1%). The administration of refralon at a maximum dose of 30 mcg/kg allowed SR to recover in 96.7% of patients, which significantly exceeds the effectiveness of amiodarone [24]. In addition, the use of cavutilide at a dose of 5 mcg/kg is accompanied by a lesser degree of QT interval prolongation and its rapid normalization. This makes it possible to reduce the time spent in the intensive care unit and to consider early initiation of antiarrhythmic therapy with class IC drugs to prevent early recurrence of AF/AFL [25]. 

Cavutilide’s i.v. effectiveness was not associated with the presence of HF, NYHA HF class, heart chamber sizes nor AF duration, whereas the efficacy of amiodarone and other AADs has direct dependence on these parameters [21,22,23]. Thus, according to Costabel J. P. et al., an average decrease in EF < 55% had a negative impact on vernakalant cardioversion effectiveness management of paroxysmal AF [26]. In this study, among patients with NYHA class III symptoms and reduced EF (<40%), the effectiveness of cavutilide was 100% with a mean duration of AF more than 70 days. In both groups of patients, the QT/QTc intervals were prolonged due to concentration-dependent blockade of the delayed rectifier potassium current, leading to the prolongation of the action potential repolarization phase and to the extension of refractory periods. From our results, less than 20% of the patients in both groups had a QT/QTc prolongation >500 ms. Only in one (0.4%) patient without HF did QTc interval prolongation to 560 ms result in non-sustained polymorphic ventricular tachycardia, which was probably associated with a variant of underlying primary electrical disease. However, this assumption required clarification. According to previously published studies, cavutilide’s i.v. administration did not lead to bradycardia development during a current AF episode [27].

During our follow-up, cavutilide also had no effect on ventricular rate during AF. However, 7.4% of patients without HF and 3.4% of patients in the HF group developed bradycardia with a heart rate less than 50 bpm after sinus rhythm restoration. In 3.5% and 1.7% of patients, respectively, pauses longer than 3 s were recorded after sinus rhythm recovery. Conduction disorders were asymptomatic in all cases, did not require additional interventions and resolved spontaneously. 

A possible mechanism for these bradyarrhythmias may be inhibition of sinoatrial node automatism due to long-term AF duration. This is consistent with the results of our previous study, indicating bradyarrhythmia appearances after cavutilide’s administration mainly in patients with persistent AF. Similar findings are often recorded after ECV.

## 5. Conclusions

This study demonstrated the high efficacy and safety of the antiarrhythmic drug cavutilide for pharmacological cardioversion management in patients with paroxysmal and persistent AF with various clinical forms of heart failure. 

## Figures and Tables

**Table 1:** Characteristics of the group of patients with HF and AF/AFL depending on the LVEF.

Parameter	*n*	LVEF, % Me, Q_1_–Q_3_
HFrEF, LVEF ≤ 40%	8	40 [38–40]
HFmrEF, LVEF 41–49%	6	45.2 [43–47]
HFpEF, LVEF ≥ 50%	44	57.6 [55–60]

HFrEF—heart failure with reduced ejection fraction; HFmrEF—heart failure with mildly reduced ejection fraction; HFpEF—heart failure with preserved ejection fraction; LVEF—left ventricular ejection fraction; Me—median, Q_1_—lower quartile (25); Q_3_—upper quartile (75).

**Table 2:** Baseline characteristics of patients (*n* = 332).

Characteristic	Patients with HF, *n* = 58	Patients without HF, *n* = 274	*p*
	Me [Q_1_;Q_3_]	Me [Q_1_;Q_3_]	
Age, year	69 [61;73]	63 [57;70]	0.023 *
Sex, male, *n*	28	163	0.12
Height	172 [160;180]	175 [165;182]	0.097
Weight, kg	86 [80;104]	90 [79;103]	0.848
BMI	31.2 [26.9;36.5]	29.4 [26.3;36.4]	0.123
Paroxysmal AF, *n*	13 (22%)	121 (44%)	0.002 *
Current duration of paroxysmal AF, day	1.5 [1;4.25]	2 [1;5]	0.442
Persistent AF, *n*	45 (78%)	153 (56%)	0.002 *
Current duration of persistent AF, day	70.5 [30;159]	82.5 [28;180]	0.658
CHA2DS2VASc	3 [2;4]	2 [1;3]	<0.001 *
LAV, mL	85 [75;95]	78 [70;90]	0.004 *
LVEF, %	57 [50;60]	60 [55;60]	<0.001 *
LVEDD, cm	5.1 [5;5.5]	5 [4.8;5.3]	0.128
LVESD, cm	3.3 [3;3.8]	3.3 [3;3.5]	0.117
LVMI	100 [89;109]	87 [77;97]	<0.001 *
AH, *n*	50 (86.2%)	223 (81.4%)	0.379
CAD, *n*	17 (29%)	28 (10.2%)	<0.001 *
MI, *n*	6 (10.3%)	10 (3.6%)	0.03 *
Diabetes mellitus. *n*	13 (22.4%)	28 (10.2%)	0.01 *

AH—arterial hypertension; BMI—body mass index; CAD—coronary artery disease; HF—heart failure; LAV—left atrial volume. LVMI—ventricular mass index. LVEF—left ventricular ejection fraction; LVEDD—left ventricular end-diastolic dimension; LVESD—left ventricular end-systolic dimension. LVMI—left ventricular mass index; MI-myocardial infarction. Me—median. Q_1_—lower quartile (25); Q_3_—upper quartile (75). *—*p* < 0.05.

**Table 3:** Drug therapy.

Drug Therapy	Patients with HF. *n*	Patients without HF. *n*	*p*
ACEi	22	84	0.238
ARB	18	59	0.212
Ca channel blockers	9	50	0.305
Loop diuretics	25	20	0.014 *
Beta-blockers	32	76	0.139
MRA	40	34	0.007 *
Statins	36	129	0.223
Apixaban	14	56	0.251
Rivaroxaban	29	95	0.208
Dabigatran etexilate	1	16	0.450
Warfarin	5	9	0.116
Enoxaparin sodium	8	65	0.344

ACEi—angiotensin-converting enzyme inhibitors; ARB—angiotensin (II) receptor blockers; HF—heart failure; MRA—mineralocorticoid receptor antagonist; *—*p* < 0.05.

## Data Availability

The data presented in this study are available from all authors.
